# GmSnRK1.1, a Sucrose Non-fermenting-1(SNF1)-Related Protein Kinase, Promotes Soybean Resistance to *Phytophthora sojae*

**DOI:** 10.3389/fpls.2019.00996

**Published:** 2019-08-02

**Authors:** Le Wang, Huiyu Wang, Shengfu He, Fanshan Meng, Chuanzhong Zhang, Sujie Fan, Junjiang Wu, Shuzhen Zhang, Pengfei Xu

**Affiliations:** ^1^Soybean Research Institute/Key Laboratory of Soybean Biology of Chinese Education Ministry, Northeast Agricultural University, Harbin, China; ^2^College of Agronomy, Plant Biotechnology Center, Jilin Agricultural University, Changchun, China; ^3^Soybean Research Institute of Heilongjiang Academy of Agricultural Sciences, Key Laboratory of Soybean Cultivation of Ministry of Agriculture P. R. China, Harbin, China

**Keywords:** *Glycine max*, *GmSnRK1.1*, enzymatic antioxidants, salicylic acid, *Phytophthora sojae*

## Abstract

Phytophthora root and stem rot, a destructive disease of soybean [*Glycine max* (L.) Merr.], is caused by the oomycete *Phytophthora sojae*. However, how the disease resistance mechanisms of soybean respond to *P. sojae* infection remains unclear. Previously, we showed that GmWRKY31, which interacts with a sucrose non-fermenting-1(SNF1)-related protein kinase (SnRK), enhances resistance to *P. sojae* in soybean. Here, we report that the membrane-localized SnRK GmSnRK1.1 is involved in the soybean host response to *P. sojae*. The overexpression of *GmSnRK1.1* (*GmSnRK1.1*-OE) increased soybean resistance to *P. sojae*, and the RNA interference (RNAi)-mediated silencing of *GmSnRK1.1* (*GmSnRK1.1*-R) reduced resistance to *P. sojae*. Moreover, the activities and transcript levels of the antioxidant enzymes superoxide dismutase and peroxidase were markedly higher in the *GmSnRK1.1*-OE transgenic soybean plants than in the wild type (WT), but were reduced in the *GmSnRK1.1*-R plants. Several isoflavonoid phytoalexins related genes *GmPAL*, *GmIFR*, *Gm4CL* and *GmCHS* were significantly higher in “Suinong 10” and *GmSnRK1.1*-OE lines than these in “Dongnong 50,” and were significantly lower in *GmSnRK1.1*-R lines. In addition, the accumulation of salicylic acid (SA) and the expression level of the SA biosynthesis-related gene were significantly higher in the *GmSnRK1.1*-OE plants than in the WT and *GmSnRK1.1*-R plants, moreover, SA biosynthesis inhibitor treated *GmSnRK1.1*-R lines plants displayed clearly increased pathogen biomass compared with H_2_O-treated plants after 24 h post-inoculation. These results showed that GmSnRK1.1 positively regulates soybean resistance to *P. sojae*, potentially functioning via effects on the expression of SA-related genes and increased accumulation of SA.

## Introduction

The sucrose non-fermenting-1(SNF1)-related protein kinases (SnRKs) are key factors in the regulation of energy metabolism, sugar signaling, seed germination, and seedling growth in plants, in addition to stress signaling in a diverse array of eukaryotes (Halford and Hey, 2009; Hey et al., 2010; Coello et al., 2011; Tsai and Gazzarrini, 2014). The SnRK1 subfamily comprises SnRK1.1, SnRK1.2, and SnRK1.3 (also named KIN10/AKIN10, KIN11/AKIN11, and KIN12/AKIN12, respectively), of which only *SnRK1.1* and *SnRK1.2* appear to be expressed in plants (Baena-González et al., 2007). SnRK1 is a heterotrimeric complex composed of an α-catalytic subunit, a γ subunit, and a β subunit that bridges the α and γ subunits (Polge and Thomas, 2007; Hedbacker and Carlson, 2008; Smeekens et al., 2010; Carling et al., 2012).

SnRK1 regulates carbon metabolism (Halford and Hardie, 1998; Fragoso et al., 2009; Nunes et al., 2013; Zhai et al., 2017) and responds to hormonal signals, particularly abscisic acid (ABA), providing a possible link between the hormone and sugar signaling pathways (Radchuk et al., 2006, 2010; Jossier et al., 2009; Coello et al., 2012; Tsai and Gazzarrini, 2012; Rodrigues et al., 2013). ABA negatively regulates resistance to *P. sojae* and active levels are depleted as part of the response to incompatible soybean genotypes (McDonald and Cahill, 1999; Mohr and Cahill, 2001; Asselbergh et al., 2007). Moreover, in wheat, SnRK1 is negatively regulated by ABA (Patricia et al., 2012). Moreover, SnRK1 regulates plant metabolism in response to stresses such as darkness and flooding, as well as developmental changes such as flowering, seed germination, and seedling growth (Baena-González et al., 2007; Jossier et al., 2009; Lee et al., 2009; Coello et al., 2011; Cho et al., 2012; Wu et al., 2017). In *Arabidopsis thaliana*, SnRK1 is involved in the responses to sugar and darkness by regulating the expression of stress-responsive genes and ABA signaling (Baena-González et al., 2007; Jossier et al., 2009). SnRK1 activities in rice (*Oryza sativa*) and Arabidopsis have a decisive influence on the expression of stress-inducible genes and the induction of stress-tolerance processes (Cho et al., 2012); for example, the rice protein kinase CIPK15 regulates carbohydrate catabolism and fermentation via the SnRK1A-MYBS1-mediated sugar signaling pathway, enabling rice to grow under floodwater (Lee et al., 2009). In Arabidopsis, FUS3 interacts with SnRK1.1 to regulate lateral organ development (Tsai and Gazzarrini, 2012), but also promotes dormancy and inhibits germination through cross-regulation of the ABA and gibberellin pathways (Gazzarrini and Tsai, 2015). Under low-sugar conditions, Arabidopsis SnRK1 was triggered to phosphorylate and inactivate the INDETERMINATE DOMAIN (IDD)-containing transcription factor IDD8, thereby leading to delayed flowering (Jeong et al., 2015). These discoveries show that SnRK1 coordinates the responses to a wide array of abiotic stresses (Baena-González et al., 2007; Lee et al., 2009; Cho et al., 2012; Jeong et al., 2015). Relatively little is known about the mechanisms by which SnRK1 functions in the responses to biotic stress. The overexpression of *SnRK1* in tobacco (*Nicotiana* sp.) made the transgenic plants more resistant to geminivirus infection (Hao et al., 2003). SnRK1 interacts with the effector AvrBsT, which is involved in suppression of the AvrBs1-specific hypersensitive response in pepper (*Capsicum annuum*) plants (Szczesny et al., 2010). The rice SnRK1b gene *OSK35* was enhanced the plant resistance to fungal and bacterial pathogens (Kim et al., 2015). Despite these insights, no systematic research on the disease-related roles of SnRK1 in another major crop species, soybean (*Glycine max*), has been reported.

In a previous study, we showed that a novel WRKY transcription factor, *GmWRKY31*, enhances soybean resistance to *P. sojae*, and identified 19 putative GmWRKY31-interacting proteins (Fan et al., 2017), of which a Sucrose non-Fermenting-1-Related Protein Kinase (SnRK1) was selected for further study. In the present study, we isolated a GmWRKY31-interacting GmSnRK1.1 (GenBank accession no. XM_006585690), and generated transgenic soybean plants either overexpressing *GmSnRK1.1* (*GmSnRK1.1*-OE) or with an RNA-interference (RNAi)-mediated reduced expression of this gene (*GmSnRK1.1-*R). Overexpression and RNA interference analysis demonstrates that GmSnRK1.1 positively regulates of soybean resistance to this pathogen, likely via a SA-signaling pathway.

## Materials and Methods

### Plant Materials and Growth Conditions

The soybean cultivar used for the various treatments and gene isolation was “Suinong 10,” which is highly resistant to *P. sojae* race 1 (PSR01) isolated in Heilongjiang, China (Zhang et al., 2010). The susceptible soybean cultivar “Dongnong 50” was used for the gene transformation experiments. These lines were obtained from the Key Laboratory of Soybean Biology in the Chinese Ministry of Education, Harbin. PSR01 was previously isolated from infected soybean plants in Heilongjiang, China (Zhang et al., 2010). This isolate was propagated at 25°C for 7 days on V8 juice agar in a glass dish. The seeds of “Suinong 10” and “Dongnong 50” were grown at 25°C and 60% relative ambient humidity in a growth cabinet, with a 16-h light/8-h dark photoperiod. For the *P. sojae* infection, the hypocotyls of soybean cultivars “Suinong 10” and “Dongnong 50” were inoculated at the first-node stage (V1) (Fehr et al., 1971) using either zoospores of *P. sojae* or a mock inoculation with sterile water following the procedure described by Kaufmann and Gerdemann (1958), with minor modifications. The *P. sojae* zoospores were induced as described by Ward et al. (1979), and the concentration of zoospores was estimated to be about 1 × 10^5^ spores mL^–1^ using a hemacytometer. The leaves of the inoculated plants were harvested and immediately frozen in liquid nitrogen at 0, 1, 3, 6, 9, 12, 24, and 48 h after the treatment, and stored at −80°C until required for RNA extraction.

### Isolation of the GmSnRK1.1 Gene

The full-length cDNA of *GmSnRK1.1* (GenBank accession no. XM_006585690) was isolated from soybean “Suinong 10” using RT-PCR with the primers *GmSnRK1.1*-F/R (see Supplementary Table S1). The extraction of total RNA and reverse transcription were performed using TRIzol reagent (Invitrogen, China) and ReverTra Ace Kit (Toyobo, Japan). The products of the RT-PCR amplification were cloned into a pMD-18T vector (Takara Bio, Japan), transformed into *Escherichia coli* DH5α cells (TransGen Biotech, China), and sequenced by GENEWIZ (China). DNAMAN software^1^ was used for the sequence alignments, and a phylogenetic analysis of *GmSnRK1.1* was carried out using MEGA5 software. The GmSnRK1.1 protein structure was analyzed using the online program Phyre2^2^.

### qRT-PCR Analysis

A qRT-PCR analysis was performed to confirm the transcript levels of *GmSnRK1.1* using a LightCycler96 instrument (Roche, Switzerland) with a real-time PCR kit (TOYOBO, Japan). *GmEF1*β (GenBank accession no. NM_001248778) was used as the internal control (see Supplementary Table S1 for primers). The relative transcript abundance of the target gene was calculated using the 2^–ΔΔCT^ method. Three biological replications were performed for each line in each analysis.

### Yeast Two-Hybrid Assays

The coding sequence of *GmSnRK1.1* was amplified and inserted into pGADT7 (Takara Bio), after which the plasmids pGADT7-*GmSnRK1.1* and pGBKT7-*GmWRKY31* were Co-transferred into the yeast strain Y2HGold (Takara Bio). The protein-protein interactions were determined by growth on three types of medium: SD (–Trp/–Leu) medium, SD (–Trp/–Leu/–His/–Ade) medium, and SD (–Trp/–Leu/–His/–Ade/X-α-gal) medium. Yeast cells carrying the pGBKT7-53 and pGADT7-SV40 plasmids were used as the positive control, and pGADT7-*GmSnRK1.1*: pGBKT7 and pGADT7:pGBKT7-*GmWRKY31* were used as the negative control.

### Bimolecular Fluorescence Complementation (BiFC) Assays

To further evaluate the interaction between GmSnRK1.1 and GmWRKY31, a BiFC assay based on yellow fluorescent protein (YFP) was performed. To construct the vectors, the coding region of GmSnRK1.1 was cloned using the primers GmSnRK1.1-bF/R and cloned into the pSAT6-cEYFP-N1 vector. The coding region of GmWRKY31 was amplified and cloned into the pSAT6-nEYFP-N1 vector (Fan et al., 2017). The plasmids were transformed into Arabidopsis protoplasts using polyethylene glycol (PEG)-mediated transfection (Yoo et al., 2007). The GmSnRK1.1-cEYFP-N1/pSAT6-nEYFP-N1 and GmWRKY31-nEYFP-N1/pSAT6-cEYFP-N1 vector combinations were used as negative controls, GmWRKY31-YEP^N/^GmHDL56-YEP^C^ were used as positive controls (Fan et al., 2017). The transfected cells were imaged using a TCS SP2 confocal spectral microscope imaging system (Leica Microsystems, Germany). The 514 nm Ar/ArKr laser was used for YFP and Chlorophyll. YFP and Chlorophyll were excitated at 514 nm and 488 nm, respectively. The wavelength range of captured light was 530–560 nm for YFP, and 650–750 nm for Chlorophyll.

### Pull-Down Assays

*GmSnRK1.1* was cloned into the pET29b (+) expression vector (Merck Millipore, United States), while *GmWRKY31* was cloned into the pGEX-4T-1 expression vector (GE Healthcare, United States). The His-GmSnRK1.1 and glutathione S-transferase-GmWRKY31 proteins were separately produced in *E. coli* BL21 (DE3) cells, then harvested and purified using a GST-Sefinose kit (Sangon, China) or a His-bind Purification Kit (Merck Millipore). The pull-down assay was performed as described by Yang et al. (2008), with minor modifications. In a total volume of 1 mL GST binding buffer (Sangon), the GST or GmWRKY31-GST recombinant proteins were incubated for 1 h at 4°C with 400 μL GST resin (Sangon), after which equal volumes of the GmSnRK1.1-His recombinant protein were added and incubated for 6 h at 4°C. The binding reaction was washed five times with binding buffer, each for 10 min at 4°C, then the pulled-down proteins were eluted by boiling, separated on a 12% SDS-PAGE gel, and immunoblotted with anti-His antibody and anti-GST antibody (Abmart, United States).

### Subcellular Localization Assays of the GmSnRK1.1 Protein

The full-length *GmSnRK1.1* sequence was cloned using RT-PCR with the primers GmSnRK1.1GF and GmSnRK1.1GR (listed in Supplementary Table S1). The coding sequence under the control of the constitutive CaMV *35S* promoter was fused to the N-terminus of the green fluorescent protein (GFP). The resulting *35S:GmSnRK1.1-GFP* expression plasmid (or the *35S:GFP* control) was transformed into Arabidopsis protoplast cells using a PEG-mediated transfection, as described by Yoo et al. (2007). The fluorescence signal was mapped using a TCS SP2 spectral confocal microscopic imaging system (Leica Microsystems). The 514 nm Ar/ArKr laser was used for GFP and Chlorophyll. GFP and Chlorophyll were excitated at 488 nm. The wavelength range of captured light was 500–530 nm for GFP, and 650–750 nm for Chlorophyll.

### Vector Construction and Transformation of Soybean

For the generation of the overexpression lines, a 4 × myc sequence was synthesized (GENEWIZ) and inserted into a pCAMBIA3301 vector to generate a pCAMBIA3301-4 × Myc plasmid, the *GmSnRK1.1* coding sequence was inserted into the *Bgl*II/*Spe*I site of the plasmid, and the 4 × myc and *bar* sequences were later used as markers. The *GmSnRK1.1* cDNA fragment was amplified using the primers *GmSnRK1.1-*R-F/R and inserted into the vector PFGC5941 (Kerschen et al., 2004). The constructs were transformed into *Agrobacterium tumefaciens* (strain LBA4404) using the freeze-thaw method (Holsters et al., 1978). “Dongnong 50” was previously used for *Agrobacterium*-mediated genetic transformation (Paz et al., 2004). Transgenic soybean plants were preliminarily verified using a PCR amplification and qPCR analysis, after which a Western blot with an anti-myc antibody (Abmart) was used to identify the plants overexpressing *GmSnRK1.1*.

### Assessment of Pathogen Resistance and the Disease Response

For pathogen infection, the living cotyledons of the WT and transgenic soybean plants at the V1 stage of development were infected with *P. sojae* zoospores (approximately 1 × 10^5^ spores mL^–1^) using the methods described by Morrison and Thorne (1978), and the roots inoculation was performed using the procedure described by Zhang et al. (2017). The disease symptoms on the infected cotyledons and roots were observed and photographed with a Nikon D7000 camera. ImageJ^3^ was used to measure the lesions of the infected cotyledons. The *P. sojae* biomass was quantified based on the accumulation of *P. sojae TEF1* (GenBank accession no. EU079791) in the soybean plants, relative to the levels of *GmEF1*β, as previously described by Chacón et al. (2010). The pathogen response assays were performed on three biological replicates, each with three technical replicates.

### Determination of Antioxidant Enzyme Activity

For the enzyme assays, the total proteins were extracted from approximately 0.1 g of leaves using 1 mL ice-cold 25 mM HEPES buffer (pH 7.8) containing 0.2 mM EDTA, 2 mM ascorbate, and 2% polyvinylpyrrolidone. The homogenates were centrifuged at 4°C for 15 min at 12,000 × *g*, after which the supernatants were carefully removed and used for the enzymatic activity measurements. The superoxide dismutase (SOD) and peroxidase (POD) activities were assayed as described by Wang et al. (2011).

### SA Measurement

Salicylic acid (SA) was extracted from the T_3_ *GmSnRK1.1* transgenic soybean leaves and quantified using HPLC-mass spectrometry, as previously described (Aboul-Soud et al., 2004, Pan et al., 2010).

## Results

### GmSnRK1.1 Interacts With GmWRKY31

**FIGURE 1:**
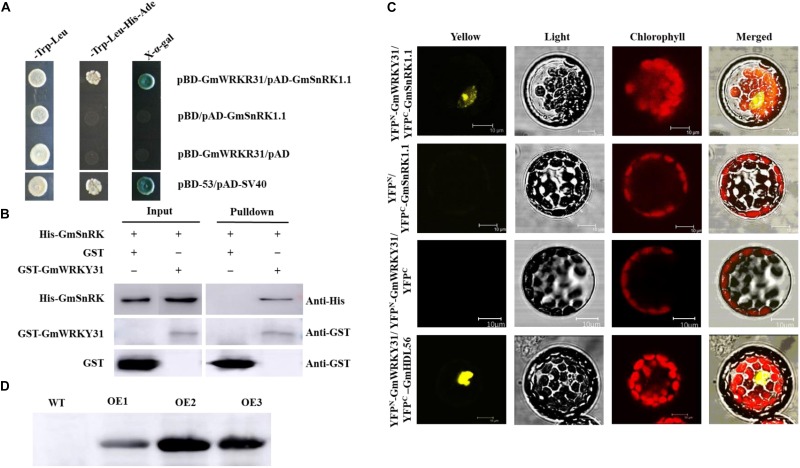
Interaction of GmSnRK1.1 with GmWRKY31 *in vitro* and *in vivo* and western blot analysis of the expression of *GmSnRK1.1*. **(A)** Analysis of interactions between GmSnRK1.1 and GmWRKY31 protein in yeast cells. The yeast cells of strain Y2H harboring pAD-GmSnRK1.1 and pBD-GmWRKY31 plasmid combinations were grown on either SD/-Trp/-Leu media or SD/-Trp/-Leu/-His/-Ade media, followed by b-galactosidase assay (SD/-Trp/-Leu/-His/-Ade/X-a-gal media). **(B)** Bimolecular fluorescence complementation (BiFC) analysis of interaction between GmSnRK1.1 and GmWRKY31 in Arabidopsis protoplast cells. The plasmid combinations are indicated on top. The fluorescence of YFP was observed by confocal laser microscopy 16 h after transfection. Bars, 10 μm. **(C)** Pull-down assay of GmSnRK1.1 interaction with GmWRKY31. His-GmSnRK1.1 protein was incubated with immobilized GST or GST-GmWRKY31 proteins, and immunoprecipitated fractions were detected by anti-His antibody. **(D)** western blot analysis of the expression of *GmSnRK1.1* in three positive overexpressing transgenic soybean lines (OE1, OE2 and OE3).

Using yeast two-hybrid assays, GmWRKY31 was found to interact with GmSnRK1.1 (Figure 1A), which was further confirmed using a BiFC assay demonstrating that GmSnRK1.1 can interact with GmWRKY31 in the nuclei of Arabidopsis protoplast cells (Figure 1C). In accordance with the results of the BiFC assay, a glutathione S-transferase pull-down assay showed that the His-tagged GmSnRK1.1 recombinant protein was pulled down by GST-GmWRKY31, but not by GST alone (Figure 1B), further indicating that GmWRKY31 interacts with GmSnRK1.1 *in vitro*. These results suggest that GmWRKY31 directly interacts with GmSnRK1.1.

### Sequence Analysis of GmSnRK1.1

The full-length GmSnRK1.1 cDNA is 1,990 bp long and contains a 1,533 bp open reading frame, which encodes a polypeptide of 510 amino acids (Supplementary Figure S2). Phylogenetic tree and alignment analyses revealed that GmSnRK1.1 shares 67.91–93.02% identity in overall amino acid sequence with its other plant species homologs, including *Lotus japonicus* LjSnRK (BAD95888), *Manihot esculenta* MeSnRK (XP_021604368), *Fragaria vesca* FvSnRK (XP_004304271), *Cucumis sativus* CsSnRK (XP_004145003), *Vitis vinifera* VvSnRK (XM_002283963.1), *Cucumis melo* CmSnRK (XP_008460108), *Pyrus bretschneideri* PbSnRK (XP_009360590), *Populus trichocarpa* PtSnRK (XP_002306053), *Morus notabilis* MnSnRK (XP_024016886), *Vicia faba* VfSnRK (AJ971809.1), *Pisum sativum* PsSnRK (CAI96819.1), *Nicotiana attenuate* NaSnRK (AAS18877), *Populus euphratica* PeSnRK (XP_011010304), *Arabidopsis thaliana* AtSnRK (M93023.1), *Daucus carota* DcSnRK (XP_017242374), *Sorghum bicolor* SbSnRK (EF544393.1), *Zea mays* ZmSnRK (AY486125.1), *Solanum tuberosum* StSnRK (CAA65244.1), *Solanum lycopersicum* SlSnRK (NP_001234325.1), and GmSnRK1.1 has the highest similarity with LjSnRK (Supplementary Figures S1B,C). The structure of GmSnRK1.1 was analyzed using Phyre, predicting that it functions as a heterotrimer complex, in which the catalytic α subunit combines with a β regulatory subunit and an activating γ subunit (Supplementary Figure S1C).

### GmSnRK1.1 Expression Is Significantly Induced by *P. sojae*

**FIGURE 2:**
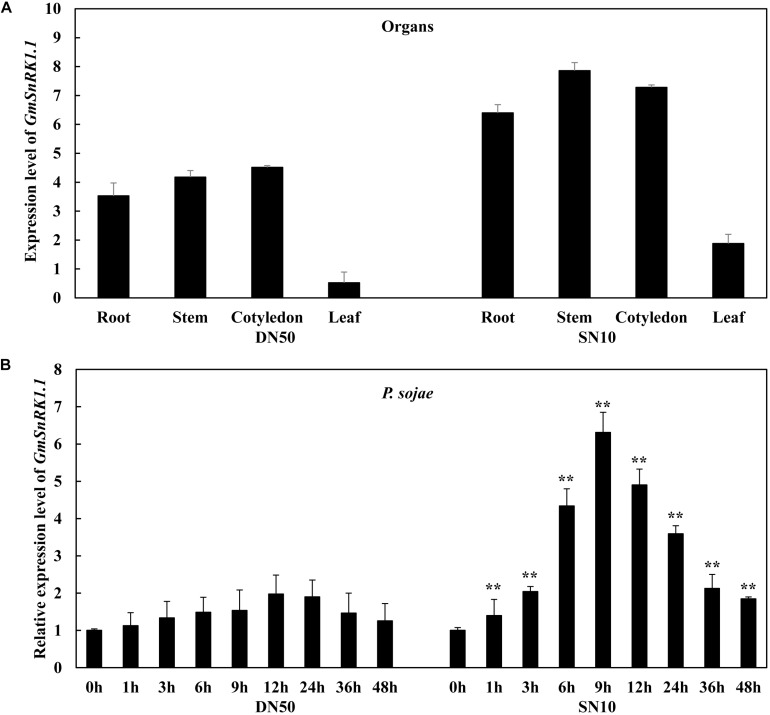
Expression patterns of *GmSnRK1.1* in *Phytophthora sojae*-resistant and *P. sojae*-susceptible soybean cultivars. **(A)** The tissue-specific expression patterns of *GmSnRK1.1* in resistant cultivar “Suinong 10” (SN10) and susceptible cultivar “Dongnong 50” (DN50) under normal conditions. **(B)** Relative expression of *GmSnRK1.1* in soybean cultivars ‘Suinong 10” and “Dongnong 50” on *P. sojae* infection. The infected samples were collected at 0, 1, 3, 6, 9, 12, 24, 36 and 48 h after *P. sojae* infection. The relative expression levels of *GmSnRK1.1* were compared with those of mock-treated plants (plants treated with sterile water) at the same time point. Fourteen-day-old soybean plants were used for analysis. The housekeeping gene of soybean *GmEF1*β (NM_001248778) was used as an internal control to normalize the data. The experiment was performed on three biological replicates, each with three technical replicates, and was statistically analyzed using Student’s *t*-test (^∗^*P* < 0.05, ^∗∗^*P* < 0.01). Bars indicate the standard error of the mean.

To evaluate the responsiveness of *GmSnRK1.1* to biotic stresses in the “Dongnong 50” and “Suinong 10” soybean cultivars, its temporal and spatial patterns were investigated using qRT-PCR. The examination of the tissue-specific transcript levels in these cultivars revealed that *GmSnRK1.1* was highly expressed in the stems, followed by the roots and cotyledons (Figure 2A). In “Suinong 10” plants inoculated with *P. sojae*, the *GmSnRK1.1* mRNA levels increased to a peak level at 9 h after inoculation, followed by a decline (Figure 2B). A similar pattern was observed in “Dongnong 50,” although the relative expression level of *GmSnRK1.1* was significantly higher in “Suinong 10” than in “Dongnong 50” (Figure 2B).

### Subcellular Localization of the GmSnRK1.1 Protein

**FIGURE 3:**
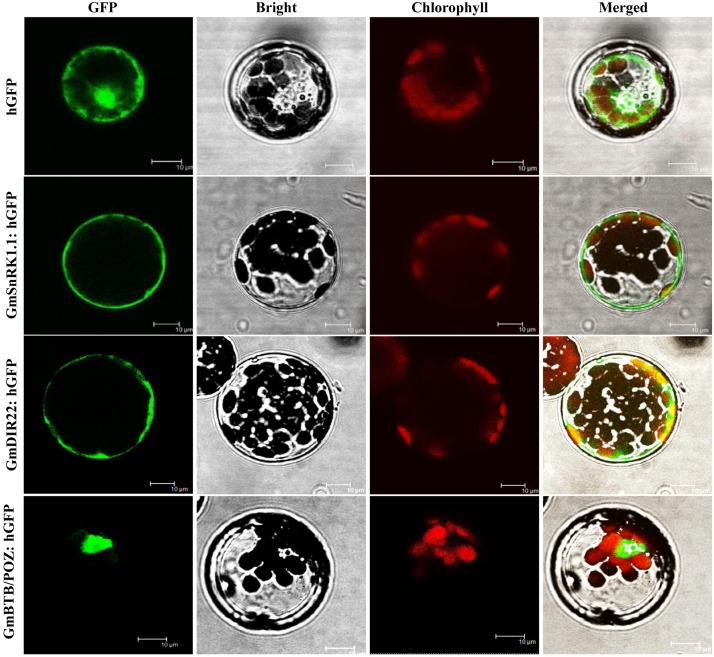
Analysis of the subcellular localization of GmSnRK1.1-GFP protein in Arabidopsis protoplasts. Subcellular localization was investigated in Arabidopsis mesophyll protoplasts under a confocal microscope. The fluorescent distribution of humanized hGFP, the fusion protein GmSnRK1.1-hGFP, GmDIR22-hGFP and GmBTB/POZ-hGFP were observed under white light, UV light, and red light, respectively. Bars, 10 μm.

The subcellular localization of the GmSnRK1.1 protein was analyzed in Arabidopsis protoplasts producing a GmSnRK1.1-GFP fusion protein under the control of the *35S* promoter. As shown in Figure 3, GFP fluorescence was distributed throughout the cells expressing the *35S:GFP* control plasmid. In contrast, the GmSnRK1.1-GFP fusion protein was exclusively localized to the Arabidopsis cell membrane, resembling the pattern of the membrane-localized GmDIR22-GFP fusion protein (Li et al., 2017) used as a control. These results indicated that GmSnRK1.1 is a membrane-localized protein.

### GmSnRK1.1 Enhances Resistance to *P. sojae* in Transgenic Soybean Plants

**FIGURE 4:**
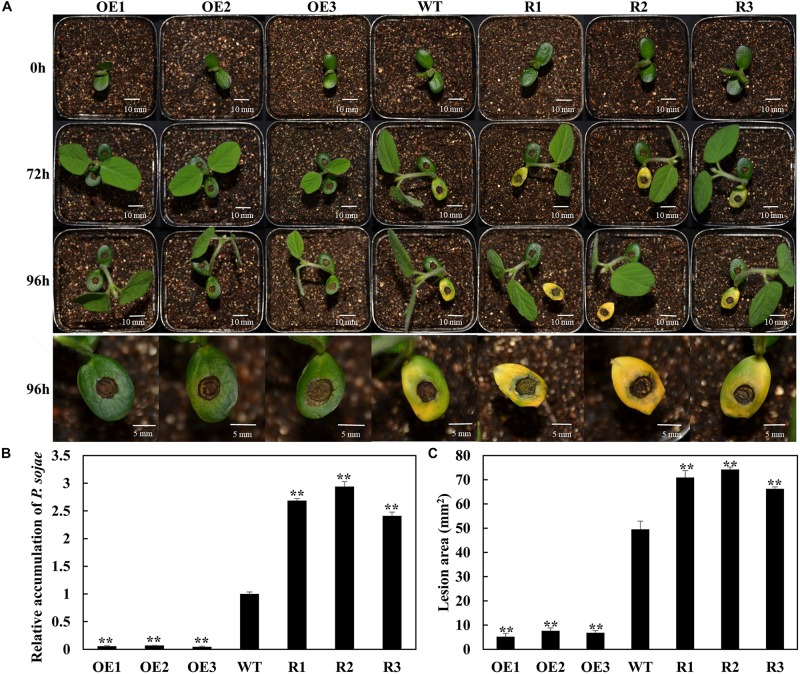
*GmSnRK1.1* enhances resistance to *Phytophthora sojae* in transgenic soybean cotyledons. **(A)** Disease symptoms on living cotyledons of *GmSnRK1.1*-overexpressing (*GmSnRK1.1*-OE), *GmSnRK1.1* RNA interference (RNAi)-mediated silencing *(GmSnRK1.1-R)* and wild-type (WT) plants at 96 h after inoculation with *P. sojae*. **(B)** Quantitative reverse transcription-polymerase chain reaction (RT-PCR) analysis of the relative biomass of *P. sojae* in *GmSnRK1.1*-OE, *GmSnRK1.1-R* transgenic lines and WT soybean based on *P. sojae TEF1* transcript levels. The experiment was performed on three biological replicates, each with three technical replicates, and statistically analyzed using Student’s *t*-test (^∗^*P* < 0.05,^∗∗^*P* < 0.01). Bars indicate the standard error of the mean. **(C)** Lesion size measured from photographed cotyledons of *GmSnRK1.1*-OE, *GmSnRK1.1-R* transgenic and WT plants at 96 h post-inoculation (hpi). The lesion size of each independent soybean line (*n* = 3) was calculated, and the lesion sizes are shown in the columns based on a comparison with the average lesion area on WT soybean.

**FIGURE 5:**
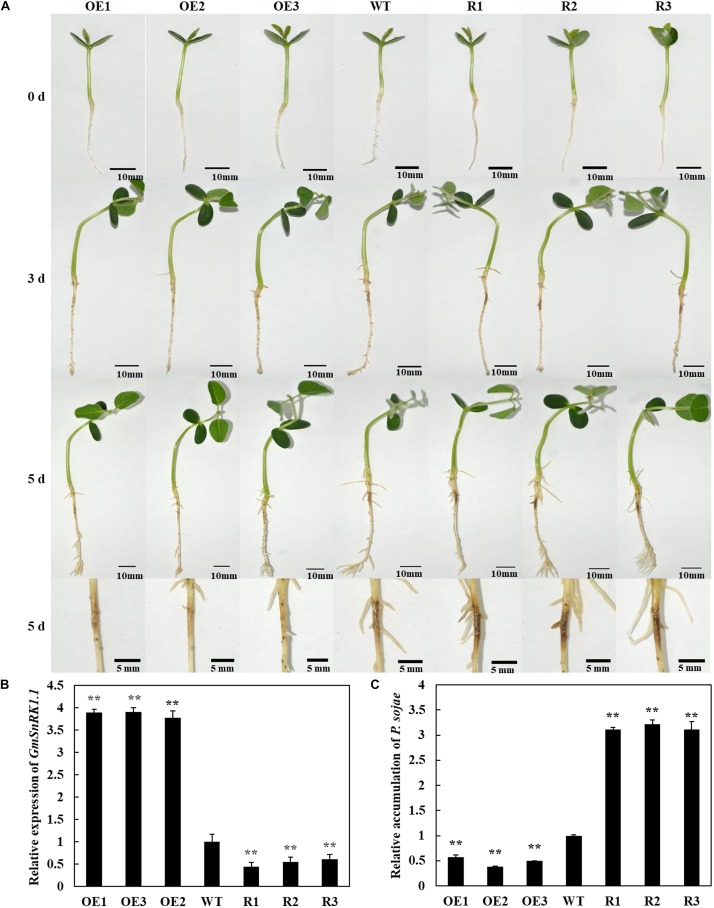
Resistance analysis of *GmSnRK1.1* transgenic soybean plants. **(A)** Disease symptoms on the roots of *GmSnRK1.1*-overexpressing (*GmSnRK1.1*-OE), *GmSnRK1.1* RNA interference (RNAi)-mediated silencing *(GmSnRK1.1-R)* and wild-type (WT) plants at 6 days after inoculation with *Phytophthora sojae*. **(B)** Quantitative reverse transcription-polymerase chain reaction (RT-PCR) analysis of the relative expression of *GmSnRK1.1* in *GmSnRK1.1*-OE, *GmSnRK1.1-R* transgenic lines and WT soybean. **(C)** Quantitative reverse transcription-polymerase chain reaction (RT-PCR) analysis of the relative biomass of *P. sojae* in *GmSnRK1.1*-OE, *GmSnRK1.1-R* transgenic lines and WT soybean based on *P. sojae* TEF1 transcript levels. The experiment was performed on three biological replicates, each with three technical replicates, and statistically analyzed using Student’s *t*-test (^∗^*P* < 0.05,^∗∗^*P* < 0.01). Bars indicate the standard error of the mean.

To analyze the function of *GmSnRK1.1* in response to infection by *P. sojae*, we generated *GmSnRK1.1*-OE and *GmSnRK1.1*-R transgenic soybean plants, which were developed into transgenic T_3_ lines. the expression of *GmSnRK1.1* in three positive overexpressing transgenic soybean lines using Western blot (Figure 1D). The resistance of the T_3_ transgenic plants to *P. sojae* was tested in their cotyledons and roots. A notable difference was observed in the development of disease symptoms after a 96 h incubation with zoospores of *P. sojae*. In the *GmSnRK1.1*-R lines, the cotyledons exhibited clear water-soaked lesions and were softer than the WT, however, almost no disease symptoms were observed in the *GmSnRK1.1*-OE lines (Figure 4A). In addition, the *P. sojae* biomass (indicated by the relative abundance of *TEF1* genomic sequence per area of infected living cotyledon) was significantly (*P* < 0.01) lower in the *GmSnRK1.1*-OE lines than in the WT plants, but higher in the *GmSnRK1.1*-R lines (Figure 4B). The lesion areas of the *GmSnRK1.1*-OE lines were significantly (*P* < 0.01) smaller than that of the WT (Figure 4C), but significantly larger in the *GmSnRK1.1-*R lines. Similar results were obtained after a 6-d incubation with *P. sojae*. The living roots of the WT soybean plants and *GmSnRK1.1*-R soybean lines exhibited watery lesions and even rotting, while those of the *GmSnRK1.1*-OE lines remained healthy (Figure 5). Similar to the results of infecting living cotyledon, the biomass of *P. sojae* after 6 days of roots infection was significantly reduced in the *GmSnRK1.1*-OE lines, but significantly increased in the *GmSnRK1.1*-R lines, relative to the WT. These results indicated that overexpression of *GmSnRK1.1* in soybean plants enhances their resistance to *P. sojae* infection. In addition, we have constructed the overexpression vector of kinase-inactive GmSnRK1.1 by synthesizing the mutation sequence of phosphorylation site (Thr157Ala, Thr235Ala, Thr261Ala). Furthermore, the overexpression of kinase-inactive GmSnRK1.1, the overexpression of *GmSnRK1.1*, and vector control transgenic soybean hairy roots generated by *Agrobacterium rhizogenes*-mediated transformation will be used to investigate the effect of the GmSnRK1.1’s kinase activity on resistance to *P. sojae* in soybean. The results demonstrated that GmSnRK1.1’s kinase activity could increase the resistance to *P. sojae* in soybean (Supplementary Figure S5).

### Overexpression of GmSnRK1.1 Affects Antioxidant Enzyme Activity

**FIGURE 6:**
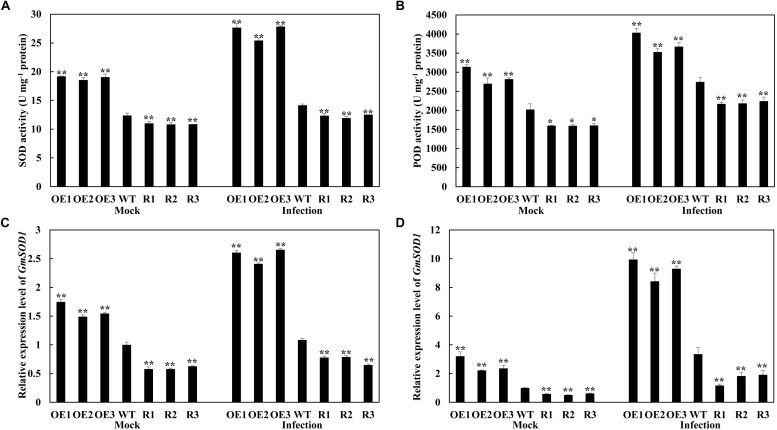
Analysis of antioxidant enzyme activity **(A,B)** and the relative expression of genes **(C,D)** under mock treatment and infected by *Phytophthora sojae* at 24 h post-inoculation (hpi). The activity of the control sample [mock-treated wild-type (WT) plants] was set to unity. The experiment was performed on three biological replicates, each with three technical replicates, and statistically analyzed using Student’s *t*-test (^∗^*P* < 0.05, ^∗∗^*P* < 0.01). Bars indicate the standard error of the mean. POD, peroxidase; SOD, superoxide dismutase.

The antioxidant defense system is well-developed in plants, involving the scavenging of reactive oxygen species (ROS) by SOD and POD (Du et al., 2001). We analyzed the SOD and POD activities in the transgenic and WT soybean plants inoculated with *P. sojae*, as well as the expression of the associated genes *GmSOD1* (NM_001248369) and *GmPOD* (XM_006575142). Under both the mock treatment and at 24 h after inoculation with *P. sojae*, the activity levels of SOD and POD, as well as the transcript abundance of the associated genes, were significantly higher in the *GmSnRK1.1*-OE lines than in the WT, but were significantly reduced in the *GmSnRK1.1*-R lines (Figure 6). These results suggested that *GmSnRK1.1* increases the activities of the antioxidant enzymes in soybean plants in response to *P. sojae* infection.

### GmSnRK1.1 Regulates the Expression of Defense-Associated Genes in Response to *P. sojae* Infection

**FIGURE 7:**
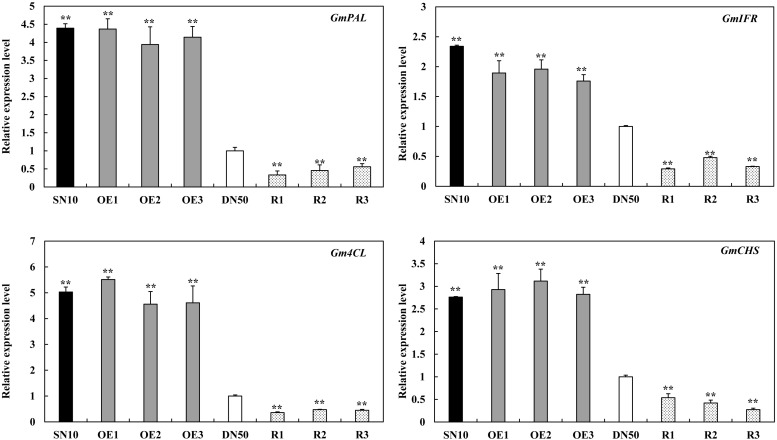
The relative transcript levels of isoflavonoid phytoalexins related genes in “Suinong 10” (SN10), “Dongnong 50” (DN50) and *GmSnRK1.1* transgenic soybean plants. The housekeeping gene of soybean *GmEF1*β was used as an internal control to normalize the data. The experiment was performed on three biological replicates with their respective three technical replicates and statistically analyzed using Student’s *t*-test (^∗^*P* < 0.05, ^∗∗^*P* < 0.01). Bars indicate the standard error of the mean.

Race-specific resistance to *P. sojae* has been shown to be mediated by isoflavonoid phytoalexins in other soybean varieties (Subramanian et al., 2005; Graham et al., 2007; Cheng et al., 2015; Zhang et al., 2017). We measured the expressions of several isoflavonoid phytoalexins genes, including *GmPAL* (GenBank accession no. NM_001250027), *GmIFR* (GenBank accession no. NM_001254100), *Gm4CL* (GenBank accession no. NM_001256363.1) and *GmCHS* (GenBank accession no. XM_003518780). These results indicated that isoflavonoid phytoalexins related genes were significantly higher in “Suinong 10” and *GmSnRK1.1*-OE lines than these in “Dongnong 50,” and were significantly lower in *GmSnRK1.1*-R lines (Figure 7). Moreover, we next monitored the expression levels of *GmSnRK1.1* during *P. sojae* infection using qRT-PCR. The transcript levels of *GmSnRK1.1* were significantly higher in the *GmSnRK1.1*-OE plants than in the WT under both the mock treatment and a 24 h infection with *P. sojae*, but were significantly lower in the *GmSnRK1.1-*R lines (Supplementary Figure S4). Pathogen-related proteins are key members in the plant response to pathogen infection (Van Loon and Van Strien, 1999; Loon et al., 2006; Xu et al., 2014). To explore the possible mechanisms of the *GmSnRK1.1*-regulated resistance to *P. sojae*, we detected the transcriptional levels of various defense-response genes, including *GmWRKY31*, *GmNPR1* (GenBank accession no. NM_001251745.1), *GmPR1* (GenBank accession no. AF136636), and *GmPR5* (GenBank accession no. M21297). As shown in Supplementary Figure S4, after 12 h incubation with *P. sojae*, the expression levels of these resistance-related genes were significantly more highly expressed in the *GmSnRK1.1*-OE plants than in the WT, but were significantly lower in the *GmSnRK1.1*-R plants. In contrast, no significant differences in the expression of *GmPR10* (GenBank accession no. FJ960440) were detected between these lines. These results indicate that one mechanism by which *GmSnRK1.1* enhances soybean defense against *P. sojae* is by regulating the expression of the defense-related genes.

### GmSnRK1.1 Affects SA Accumulation and the Expression of the SA Biosynthesis Genes

**FIGURE 8:**
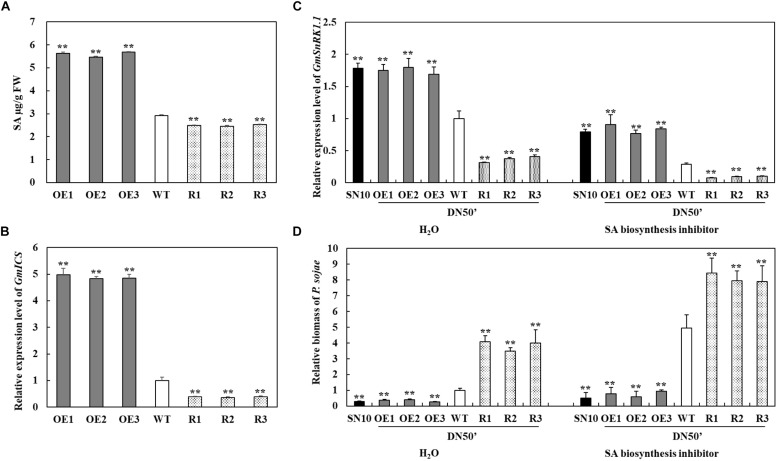
Investigation of the relationship between *GmSnRK1.1* and the salicylic acid (SA) pathway in soybean. **(A)** SA contents in leaves of transgenic and wild type (WT) soybean. FW, fresh weight. **(B)** Relative transcript level of *GmICS* in *GmSnRK1.1*-overexpressing (*GmSnRK1.1*-OE), *GmSnRK1.1* RNA interference (RNAi)-mediated silencing *(GmSnRK1.1-R)* transgenic and WT soybean. The level of the control sample (WT plants) was set to unity. **(C)** Relative transcript level of *GmSnRK1.1* in “Suinong 10” (SN10), “Dongnong 50” (DN50) and *GmSnRK1.1* transgenic soybean lines treated with H_2_O (mock) and SA biosynthesis inhibitor (100 μM ABT) under *P. sojae* infection. **(D)** The relative biomass of *P. sojae* in “Suinong 10” (SN10), “Dongnong 50” (DN50) and *GmSnRK1.1* transgenic soybean lines treated with H_2_O (mock) and SA biosynthesis inhibitor (100 μM ABT) under *P. sojae* infection. The experiment was performed on three biological replicates, each with three technical replicates, and was statistically analyzed using Student’s *t*-test (^∗^*P* < 0.05, ^∗∗^*P* < 0.01). Bars indicate the standard error of the mean.

To test whether the *GmSnRK1.1* could regulate the accumulation of SA, the SA contents of the T_3_ *GmSnRK1.1* transgenic lines were evaluated. As shown in Figure 8A, the *GmSnRK1.1*-OE transgenic soybean leaves contained significantly more SA than the WT leaves, while the *GmSnRK1.1*-R plants accumulated significantly less SA. we also analyzed the transcript levels of *GmICS1* (XM_003522145), which plays a key role in SA biosynthesis. *GmICS1* was significantly more highly expressed in the *GmSnRK1.1*-OE transgenic lines than in the WT, while the *GmSnRK1.1*-R transgenic lines has a significantly lower level of *GmICS1* expression (Figure 8B). Furthermore, whether or not the defense mechanism is dependent on SA, we measured the expression level of *GmSnRK1.1* and the relative biomass of *P. sojae* in “Suinong 10,” “Dongnong 50” and *GmSnRK1.1* transgenic soybean lines treat with sterile water and SA biosynthesis inhibitor (100 μM 1-aminobenzotriazole). As expected, SA biosynthesis inhibitor treated plants displayed clearly increased pathogen biomass compared with H_2_O-treated plants after 24 h post-inoculation (Figure 8D). These results suggested that *GmSnRK1.1* plays a positive important role in the response to *P. sojae* infection, increasing the disease-resistance of soybean via a possible mechanism involving the SA signaling pathway.

## Discussion

Identification and characterization of genes involved in response to *P. sojae* infection in soybean has contributed to our understanding of the genetic mechanisms of resistance (Xu et al., 2014; Cheng et al., 2015; Kong et al., 2015; Zhang et al., 2017). In our previous study, GmWRKY31 was found to play a key role in increasing the disease resistance of soybean plants to *P. sojae* infection (Fan et al., 2017). In the present study, we identified and functionally characterized GmSnRK1.1 as an interacting partner of GmWRKY31, demonstrating that it plays a positive role in the response to *P. sojae* infection. The SnRK1 protein kinases are considered central regulators of the energy metabolism and stress signaling in plants (Baena-González and Sheen, 2008; Radchuk et al., 2010; Tsai and Gazzarrini, 2012), however, the biological functions of the SnRK1s in soybean are poorly understood. Here, we first discovered that the overexpression of *GmSnRK1.1* significantly increased the plant responses to *P*. *sojae* infection, while RNA-interference mediated reduced expression of this gene significantly increased the susceptibility of the transgenic plants to this pathogen (Figures 4, 5).

Previous studies have confirmed that the SnRK1s play a role in the plant defense against viruses, fungi, and bacteria (Hao et al., 2003; Szczesny et al., 2010; Kim et al., 2015); for example, the overexpression of *SnRK1* was shown to significantly increase the resistance of tobacco plants to geminivirus infection (Hao et al., 2003). In rice, *OSK35* (a rice *SnRK1b* gene) positively regulates the disease resistance of plants subjected to the fungal pathogen *Magnaporthe oryzae* and the bacterial pathogen *Xanthomonas oryzae* pv. *oryzae* (*Xoo*) (Kim et al., 2015). The wheat (*Triticum aestivum*) alpha subunit of TaSnRK1a interacts with TaFROG (Fusarium Resistance Orphan Gene) to increase the disease resistance response to the mycotoxigenic fungus *Fusarium graminearum* (Perochon et al., 2015). The hypersensitive response was induced by the *Xanthomonas campestris* pv. *vesicatoria* effector AvrBs1 in *snrk1* mutant pepper plants (Szczesny et al., 2010). In this work, the temporal and spatial patterns of *GmSnRK1.1* expression were analyzed in the *P. sojae*-resistant soybean cultivar “Suinong 10” and the susceptible cultivar “Dongnong 50,” revealing that *GmSnRK1.1* was markedly expressed in the stems of these lines, with lower expression levels in the roots and cotyledons (Figure 2A). We detected that the expression levels of *GmSnRK1.1* following *P. sojae* infection were much higher in “Suinong 10” than in the susceptible cultivar “Dongnong 50,” and that *P. sojae* infection markedly increased the expression levels of *GmSnRK1.1* in both cultivars (Figure 2B). In addition, *GmSnRK1.1* is negatively regulated by ABA (Supplementary Figure S3). We further demonstrated that the *GmSnRK1.1*-OE transgenic plants had an increased disease resistance to *P. sojae* infection, while the *GmSnRK1.1*-R transgenic plants exhibited increased susceptibility (Figures 4, 5).

Further research showed that *GmSnRK1.1* positively regulates the activities and transcription levels of antioxidant enzymes SOD and POD (Figure 6). SOD constitutes the first line of defense against ROS within a cell (Alscher et al., 2002), while POD plays a key role in scavenging ROS and preventing cellular damage (Tewari et al., 2006). Therefore, we further analysis the activities and transcription level of SOD and POD, In plants infected with *P. sojae*, *GmSnRK1.1* positively enhanced the activities and transcription levels of SOD and POD, enabling them to scavenge the ROS and provide sufficient protection against oxidative damage.

The phytohormones SA, jasmonic acid, and ethylene play central roles in regulating the plant responses to pathogen attack (Reymond and Farmer, 1998; Kunkel and Brooks, 2002; Spoel et al., 2003; Robert-Seilaniantz et al., 2011; Alazem and Lin, 2015). SA mediates and activates the biotic stress response to pathogenic challenge (Pieterse et al., 2009; Sugano et al., 2013; Alazem and Lin, 2015), with the transcriptional cofactor *NPR1* playing a key role in the SA-signaling pathway of several plant species (Vlot et al., 2009). In rice, OsSnRK1a positively regulates plant resistance by linking to the SA pathway (Filipe et al., 2018). In previous studies, the overexpression of *GmWRKY31* was found to induce the expression of *GmNPR1*, increasing the disease resistance of soybean plants in response to *P. sojae* infection via the activation of the SA-signaling pathway (Fan et al., 2017). The results of this study supported these findings, as the expression levels of *GmWRKY31* and *GmNPR1* were markedly higher in the *GmSnRK1.1*-OE transgenic plants in comparison with the WT and lower in the *GmSnRK1.1*-R transgenic plants (Supplementary Figure S4). A downstream member of the SnRK1 signaling pathway, *STOREKEEPER RELATED1/G-Element Binding Protein* (*STKR1*), was previously found to display transcriptional changes which constitutively activated the SA-related defense in transformed Arabidopsis plants (Nietzsche et al., 2018). We also found that the overexpression of *GmSnRK1.1* induced the expression of *GmPR1* and *GmPR5*, which are effector genes for the systemic acquired resistance response, a process mediated by SA (Ward et al., 1991; He et al., 2007). The high expression levels of these genes indicated that SA signaling was activated in the *GmSnRK1.1-*OE plants, which was confirmed by our determination that SA accumulation and the expression of the SA biosynthesis gene *GmICS1* were upregulated in these plants relative to the WT (Figure 8). In addition, we examined the expression level of *GmSnRK1.1* and relative biomass of *P. sojae* in “Suinong 10,” “Dongnong 50” and *GmSnRK1.1* transgenic soybean lines treated with H_2_O (mock) and SA biosynthesis inhibitor (100 μM ABT) under *P. sojae* infection, the results showed that SA is involved in GmSnRK1.1-mediating defense to *P. sojae* (Figure 8). These indicated that GmSnRK1.1 promotes the accumulation of SA and the expression of *GmICS1*, and suggests that GmSnRK1.1 acts as a positive regulator of the downstream defense pathways and SA-dependent defense signaling.

In Glycine soja, a ABA activated calcium independent SnRK-type kinase, GsAPK, was localized in the plasma membrane (Liang et al., 2012). In Arabidopsis, SnRK1 is localized to the plant nucleus and endoplasmic reticulum (Blanco et al., 2019). In this work, GmSnRK1.1 was localized in the plasma membrane (Figure 3), and GmWRKY31 was localized to the plant nucleus (Fan et al., 2017). The BiFC assay showed that GmSnRK1.1 interacted with GmWRKY31 in the nucleus (Figure 1), but the mechanism of nuclear interaction is not clear which require further study and discussion.

## Data Availability

All datasets generated for this study are included in the manuscript and/or the Supplementary Files.

## Author Contributions

PX and SZ designed the experiments. LW, HW, SH, and FM performed the experiments. FM, LW, SF, and JW analyzed the data. LW, PX, SZ, and CZ wrote the manuscript.

## Conflict of Interest Statement

The authors declare that the research was conducted in the absence of any commercial or financial relationships that could be construed as a potential conflict of interest.

## Footnotes

1 http://www.lynnon.com/

2 http://www.sbg.bio.ic.ac.uk/phyre2

3 https://imagej.nih.gov/ij/index.html

## Supplementary Material

FIGURE S1 Sequence comparison of GmSnRK1.1 with orthologs from other plant species. **(A)** Phylogenetic analysis of GmSnRK1.1 with orthologs from other plant species. **(B)** Alignment of amino acid sequences of GmSnRK1.1 with orthologs from other plant species. **(C)** The tertiary structure of the GmSnRK1.1 protein.

FIGURE S2 The open reading frame sequence and deduced polypeptide sequence of *GmSnRK1.1.*

FIGURE S3 The relative transcript levels of *GmSnRK1.1* at various time points post-treatment with ABA in “Suinong 10” and “Dongnong 50” soybean plants. Fourteen day-old plants were used for the treatments and analyses. The housekeeping gene of soybean *GmEF1*β was used as an internal control to normalize the data. The relative transcript levels of *GmSnRK1.1* were quantified compared with mock plants at the same time points. The experiment was performed on three biological replicates with their respective three technical replicates and statistically analyzed using Student’s *t*-test (^∗^*P* < 0.05, ^∗∗^*P* < 0.01). Bars indicate the standard error of the mean.

FIGURE S4 Relative expression levels of defense-associated genes in soybean plants under mock treatment and infected by *Phytophthora sojae* at 24 h post-inoculation (hpi). The housekeeping gene of soybean *GmEF1*β was used as an internal control to normalize the data. The expression level of the control sample [mock-treated wild-type (WT) plants] was set to unity. The experiment was performed on three biological replicates, each with three technical replicates, and was statistically analyzed using Student’s *t*-test (^∗^*P* < 0.05, ^∗∗^*P* < 0.01). Bars indicate the standard error of the mean.

FIGURE S5 The effect of GmSnRK1.1 kinase assay on the resistance to *P. sojae*. **(A)** Mutation of GmSnRK1.1 activation sites. **(B)** Disease symptoms on the hairy roots of the overexpression of GmSnRK1.1 (*GmSnRK1.1*-OE), the overexpression of kinase-inactive GmSnRK1.1 (*GmSnRK1.1* mutation-OE), and empty vector control (3301), *GmSnRK1.1* RNA interference (RNAi)-mediated silencing (*GmSnRK1.1-*R) transgenic soybean at 4 days after inoculation with *Phytophthora sojae*. **(C)** Quantitative reverse transcription-polymerase chain reaction (RT-PCR) analysis of the relative biomass of *P. sojae* in *GmSnRK1.1*-OE, *GmSnRK1.1* mutation-OE, *GmSnRK1.1-R*, and 3301 empty vector transgenic soybean hairy roots based on *P. sojae* TEF1 transcript levels. The experiment was performed on three biological replicates, each with three technical replicates, and statistically analyzed using Student’s *t*-test (^∗^*P* < 0.05, ^∗∗^*P* < 0.01). Bars indicate the standard error of the mean. **(D)** Transgenic soybean hairy roots were tested using Liberty Link strips.

TABLE S1 Primer sequences used in this study.
