# Supplementary figures and images for: GmSnRK1.1, a Sucrose Non-fermenting-1(SNF1)-Related Protein Kinase, Promotes Soybean Resistance to Phytophthora sojae
Source: Front Plant Sci. 2019 Aug 2;10:996. doi: 10.3389/fpls.2019.00996 (PMC6688127; doi:10.3389/fpls.2019.00996)

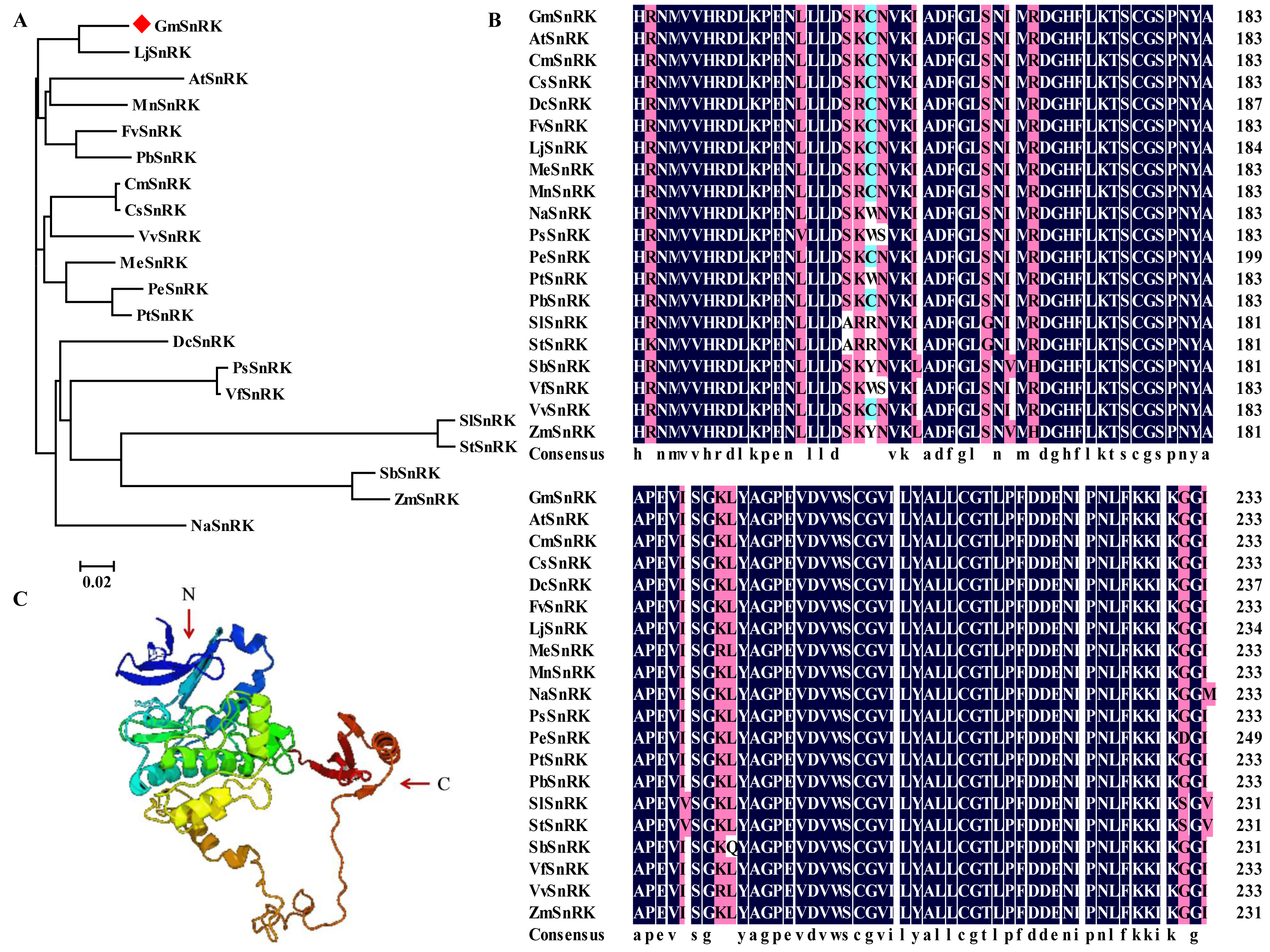

Supplement: FIGURE S1 — Sequence comparison of GmSnRK1.1 with orthologs from other plant species. (A) Phylogenetic analysis of GmSnRK1.1 with orthologs from other plant species. (B) Alignment of amino acid sequences of GmSnRK1.1 with orthologs from other plant species. (C) The tertiary structure of the GmSnRK1.1 protein. [file Image_1.JPEG]

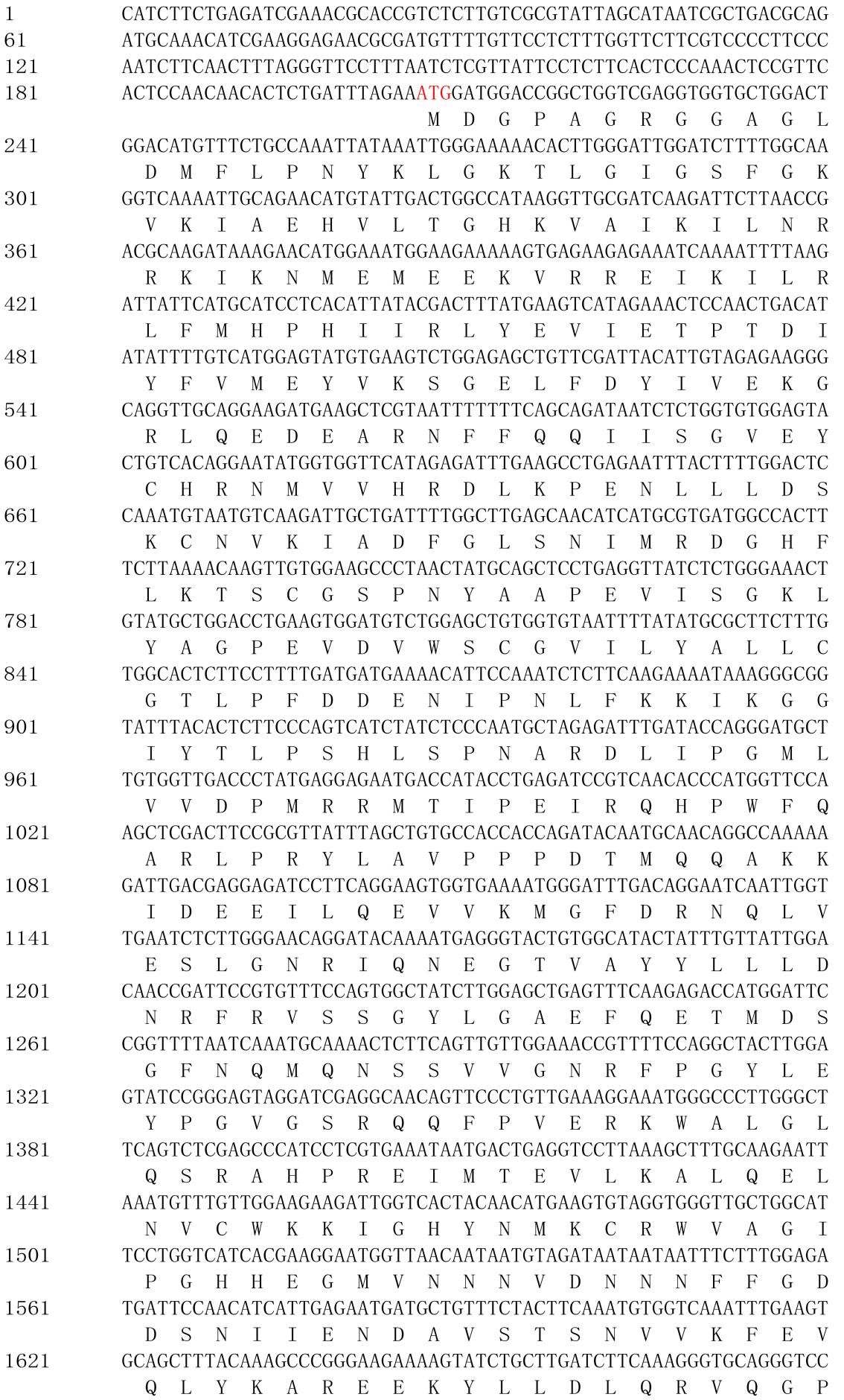

Supplement: FIGURE S2 — The open reading frame sequence and deduced polypeptide sequence of GmSnRK1.1. [file Image_2.JPEG]

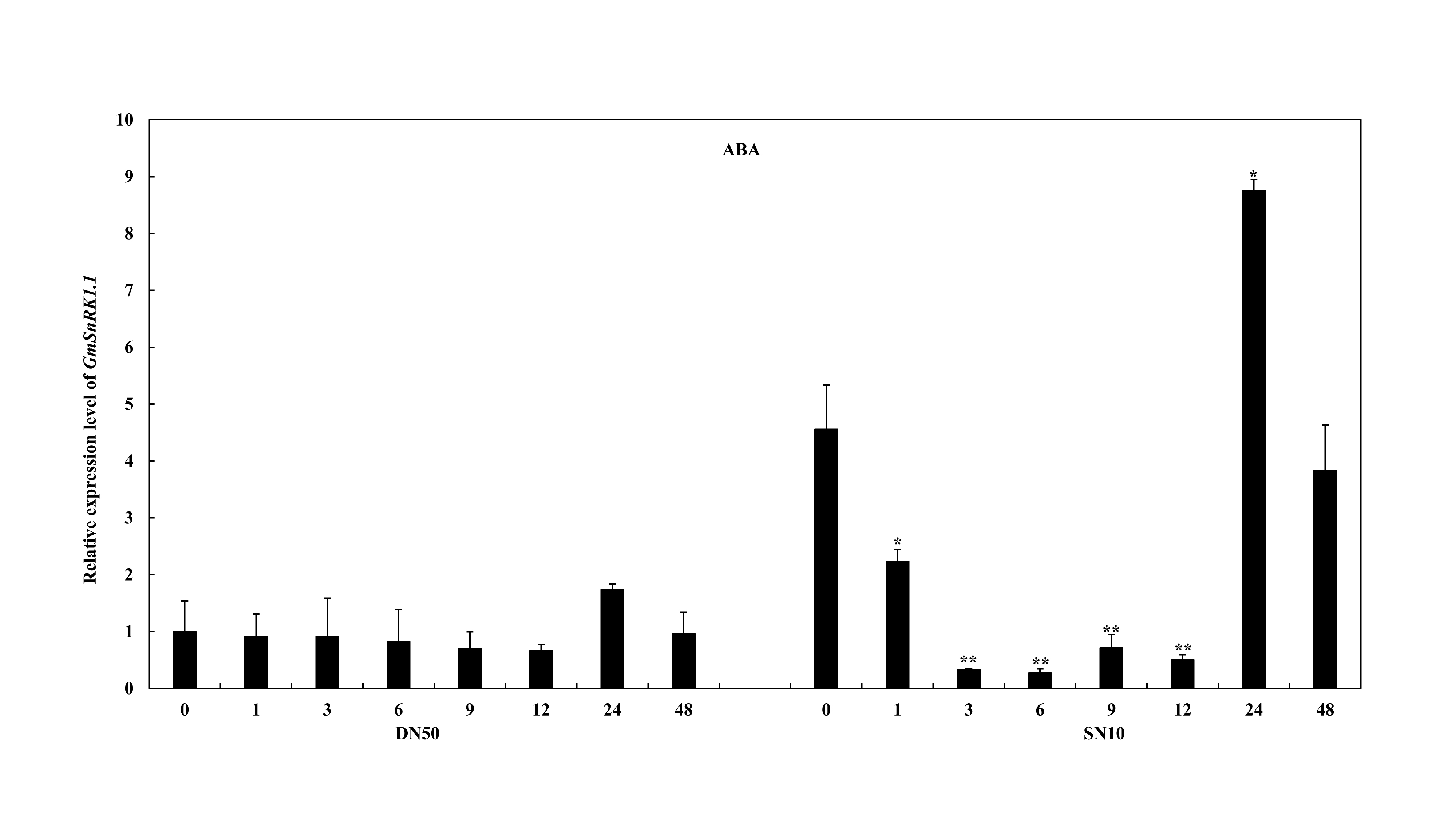

Supplement: FIGURE S3 — The relative transcript levels of GmSnRK1.1 at various time points post-treatment with ABA in “Suinong 10” and “Dongnong 50” soybean plants. Fourteen day-old plants were used for the treatments and analyses. The housekeeping gene of soybean GmEF1β was used as an internal control to normalize the data. The relative transcript levels of GmSnRK1.1 were quantified compared with mock plants at the same time points. The experiment was performed on three biological replicates with their respective three technical replicates and statistically analyzed using Student’s t-test (∗P < 0.05, ∗∗P < 0.01). Bars indicate the standard error of the mean. [file Image_3.JPEG]

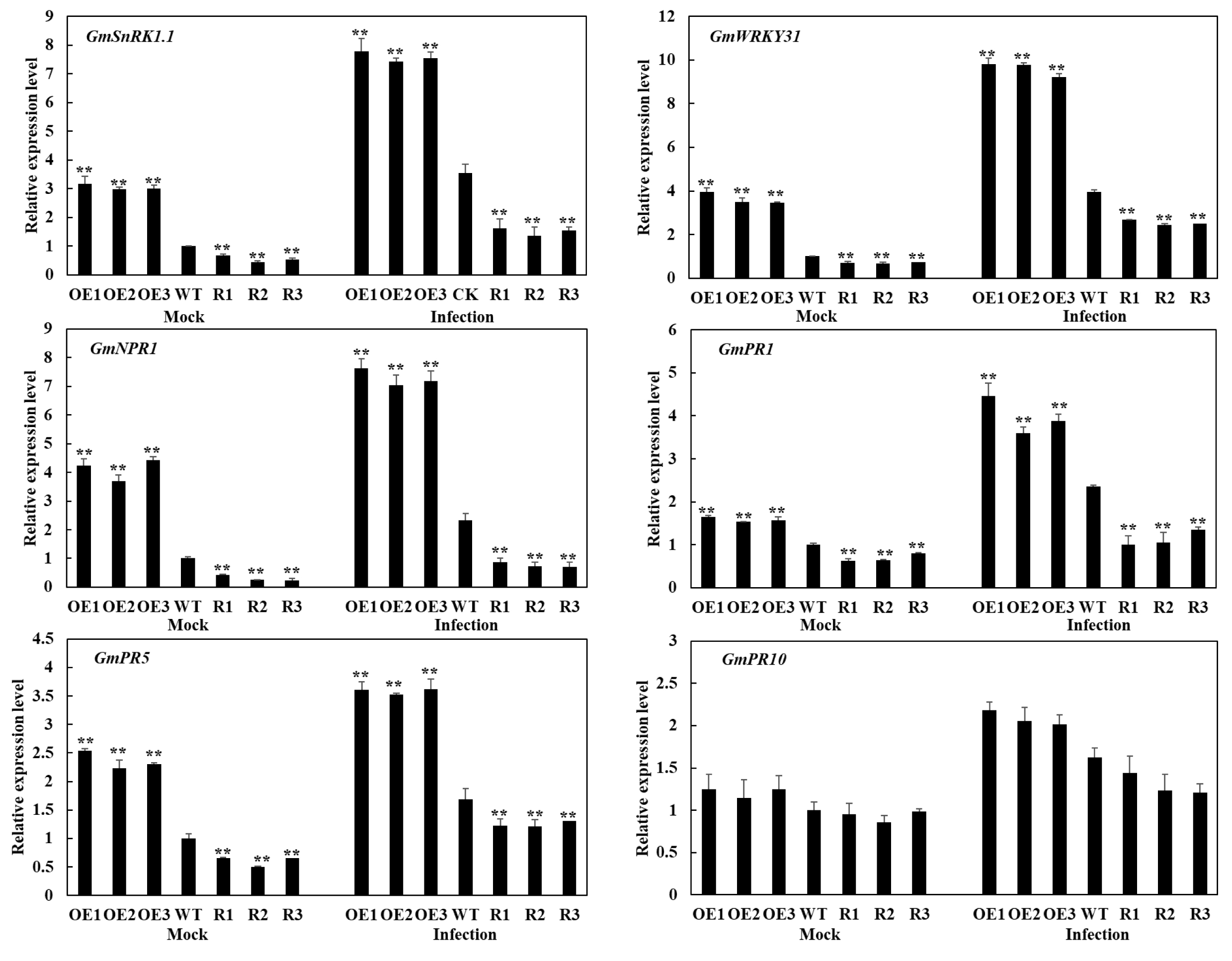

Supplement: FIGURE S4 — Relative expression levels of defense-associated genes in soybean plants under mock treatment and infected by Phytophthora sojae at 24 h post-inoculation (hpi). The housekeeping gene of soybean GmEF1β was used as an internal control to normalize the data. The expression level of the control sample [mock-treated wild-type (WT) plants] was set to unity. The experiment was performed on three biological replicates, each with three technical replicates, and was statistically analyzed using Student’s t-test (∗P < 0.05, ∗∗P < 0.01). Bars indicate the standard error of the mean. [file Image_4.JPEG]

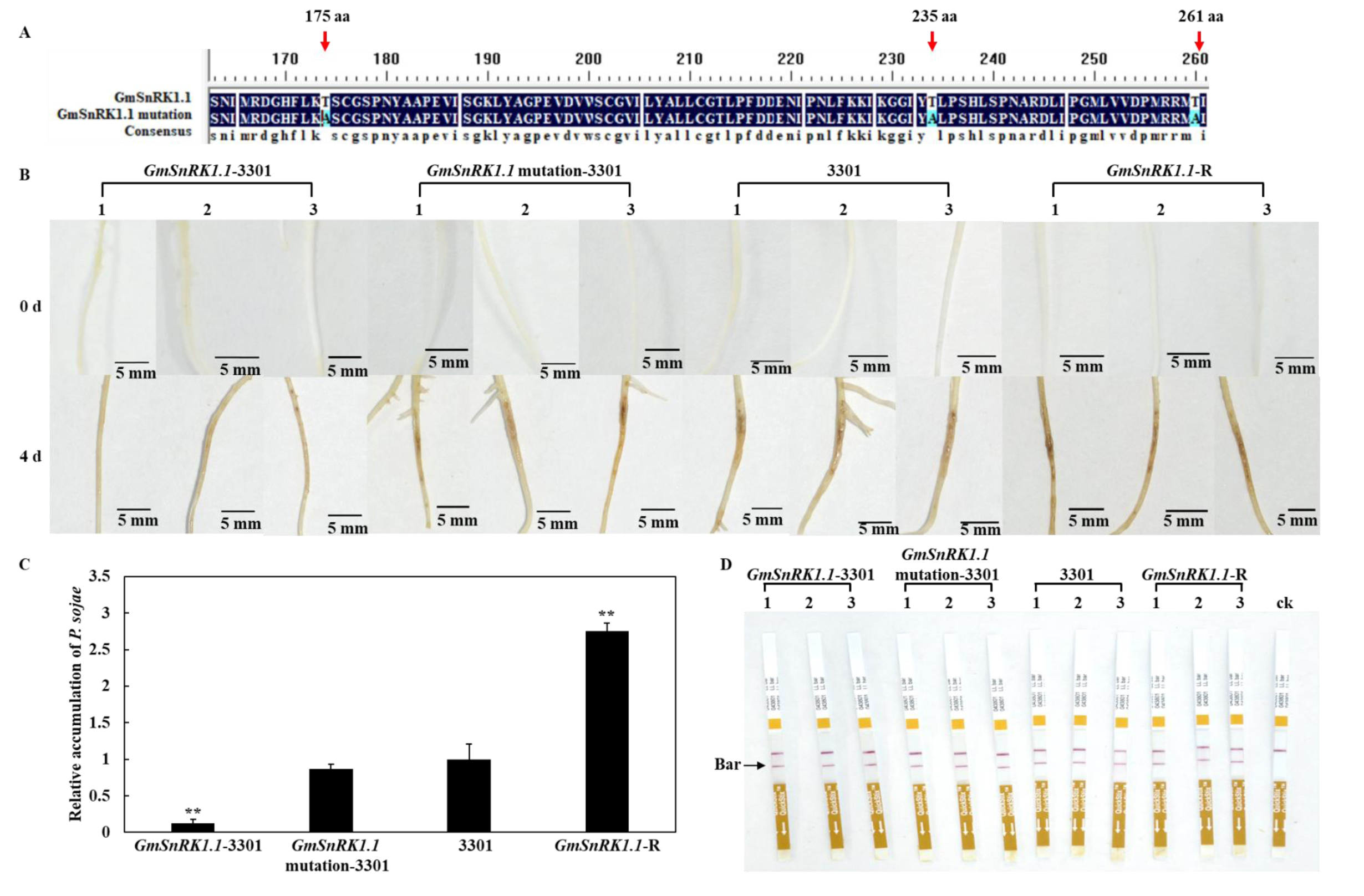

Supplement: FIGURE S5 — The effect of GmSnRK1.1 kinase assay on the resistance to P. sojae. (A) Mutation of GmSnRK1.1 activation sites. (B) Disease symptoms on the hairy roots of the overexpression of GmSnRK1.1 (GmSnRK1.1-OE), the overexpression of kinase-inactive GmSnRK1.1 (GmSnRK1.1 mutation-OE), and empty vector control (3301), GmSnRK1.1 RNA interference (RNAi)-mediated silencing (GmSnRK1.1-R) transgenic soybean at 4 days after inoculation with Phytophthora sojae. (C) Quantitative reverse transcription-polymerase chain reaction (RT-PCR) analysis of the relative biomass of P. sojae in GmSnRK1.1-OE, GmSnRK1.1 mutation-OE, GmSnRK1.1-R, and 3301 empty vector transgenic soybean hairy roots based on P. sojae TEF1 transcript levels. The experiment was performed on three biological replicates, each with three technical replicates, and statistically analyzed using Student’s t-test (∗P < 0.05, ∗∗P < 0.01). Bars indicate the standard error of the mean. (D) Transgenic soybean hairy roots were tested using Liberty Link strips. [file Image_5.JPEG]
