# Supplementary material for: GmSnRK1.1, a Sucrose Non-fermenting-1(SNF1)-Related Protein Kinase, Promotes Soybean Resistance to Phytophthora sojae
Source: Front Plant Sci. 2019 Aug 2;10:996. doi: 10.3389/fpls.2019.00996 (PMC6688127; doi:10.3389/fpls.2019.00996)
Supplement: TABLE S1 — Primer sequences used in this study. [file Table_1.DOCX]

**Table S1. The primer sequences used in this study.**

| Overexpression | GmSnRK1.1-F | GAAGATCTAATGGATGGACCGGCTG |
| --- | --- | --- |
|  | GmSnRK1.1-R | GGGTAACCCTAGAGGACCCGAAGC |
| RNAi | GmSnRK1.1-R(1)-F | CCGCTCGAGTCTTCCCAGTCATCTATCTCCC |
|  | GmSnRK1.1-R(1)-R | CATGCCATGGCCCTCATTTTGTATCCTGTTCC |
|  | GmSnRK1.1-R(2)-F | GCTCTAGATCTTCCCAGTCATCTATCTCCC |
|  | GmSnRK1.1-R(2)-R | CGGGATCCCCCTCATTTTGTATCCTGTTCC |
| Yeast two-hybrid | pGADT7-GmSnRK-F | CCGGAATTCATGGATGGACCGGCTGGT |
|  | pGADT7-GmSnRK-R | CGCGGATCCGAGGACCCGAAGCTGTGC |
|  | pGBKT7-GmWRKY31-F | GCCGAATTCATGGACAAAGGATGGGGACTC |
|  | pGBKT7-GmWRKY31-R | GGTCGACGTTTCCTGAAAAGCTGCTAATGG |
| BIFC | GmWRKY31-bF | CCGGAATTCATGGACAAAGGATGGGG |
|  | GmWRKY31-bR | CGCGGATCCAGTTTCCTGAAAAGCTG |
|  | GmSnRK-bF | CCGGAATTCATGGATGGACCGGCTGGT |
|  | GmSnRK-bR | CGCGGATCCAGAGGACCCGAAGCTGTGC |
| Pulldown | GmWRKY31-PF | CCGGGATCCATGGACAAAGGATGGGG |
|  | GmWRKY31-PR | CGCGAATTCAGTTTCCTGAAAAGCTG |
|  | GmSnRK-PF | CCGGAATTCATGGATGGACCGGCTGGT |
|  | GmSnRK-PR | CGCGTCGACAGAGGACCCGAAGCTGTGC |
| GFP | *GmSnRK1.1*-gF | GAAGATCTAATGGATGGACCGGCTGG |
|  | *GmSnRK1.1*-gR | GCACTAGTGAGGACCCGAAGCTGTGC |
| qRT-PCR | TEF1-QF | TGATCGTGCTGAACCACCC |
|  | TEF1-QR | CGAGCGACGGTCCATCTT |
|  | SnRK1.1QF | TTCCGTGTTTCCAGTGGCTAT |
|  | SnRK1.1QR | AAGGACCTCAGTCATTATTTCACG |
|  | GmICS-QF | CACGGTTCACAAGTGGCTCA |
|  | GmICS-QR | GTTGGTGTTGTCCCTCTCTGC |
|  | GmWRKY31-QF | ACCTCACACACAACCCTAACCC |
|  | GmWRKY31-QR | CCTACATCCTGAGACAACTGAAGAC |
|  | GmNPR1-QF | TCTTTGGGTTTTCGGTCT |
|  | GmNPR1-QR | CAACTTTCCTGCTTTCACA |
|  | *GmSOD1*-QF | CAGTTCTTGGCAGCAGCGA |
|  | *GmSOD1*-QR | CACCGTGCTCGTTGTTATTAGG |
|  | *GmPOD*-QF | AAGCCTCATTCGCCTCCAC |
|  | *GmPOD*-QR | CAAGATACGACTCCAGGGCAA |
|  | *GmPR1-*QF | TGAAAATGTGGGTTGATGAGAAAT |
|  | *GmPR1*-QR | AAGTGATGAAAGTGCCTCCGTT |
|  | *GmPR5*-QF | CCCTCGCCTCCACTTCTTC |
|  | *GmPR5*-QR | TTGGTGCTCATCTTGCCTCTA |
|  | GmPR10-QF | TAGCATCCACAGCATTGTTTTC |
|  | GmPR10-QR | CAAGGCAGTGCCCTCAGTTA |
|  |  |  |
|  |  |  |
